# Relationships between Employees’ Identifications and Citizenship Behavior in Work Groups: The Role of the Regularity and Intensity of Interactions

**DOI:** 10.3390/bs11070092

**Published:** 2021-06-22

**Authors:** Andrey V. Sidorenkov, Eugene F. Borokhovski

**Affiliations:** 1 Academy of Psychology and Educational Sciences, Southern Federal University, 105/42 Bolshaya Sadovaya Str., 344006 Rostov-on-Don, Russia; av.sidorenkov@yandex.ru; 2 Centre for the Study of Learning and Performance (CSLP), Concordia University, 1515 St. Catherine Street West, S-GA-2.126, Montreal, QC H3G 1W1, Canada

**Keywords:** personal identification, interpersonal identification, micro-group identification, group identification, organizational identification, organizational citizenship behavior, organizational communicativeness

## Abstract

This paper explores the relationships of various employees’ identifications (personal, interpersonal, micro-group, group and organizational) in their two components (cognitive and affective) with two dimensions of organizational citizenship behavior (OCB): offering quality ideas and suggestions, and providing help and support within small work groups. Two studies were conducted in Russia on two respective samples: (1) employees of commercial enterprises (*N* = 183) characterized by a relatively high regularity and intensity of within-group interactions; and (2) the academic staff of higher education institutions (*N* = 157), which typically have relatively less regular, low-intensity within-group interactions. The research employed four questionnaires to assess the participants’ identifications in both of their components. In addition, managers in the respective organizations filled out an organizational communicativeness questionnaire and a two-factor OCB assessment instrument. It was found that the relationships between (a) particular identifications and (b) the ratio of group identification to other identifications, on the one hand, and OCB, on the other, depend on the degree of regularity of within-group interactions, as well as on the identification components. Organizational communicativeness did not moderate the relationship between identifications and OCB, but was significantly positively correlated with both OCB dimensions. The theoretical and practical implications of the study findings are discussed.

## 1. Introduction

Researchers and practitioners have consistently focused their interest on organizational citizenship behavior [1,2] and related forms of behavior, such as contextual performance [3,4], extra-role behavior [5] and organizational spontaneity [6]. The constructs that reflect these and similar forms of behavior intersect with each other, which is partly responsible for the fact that the issue of their distinctive features has not been fully resolved [7]. The term “Organizational Citizenship Behavior” (OCB) will be used here in a broad sense, encompassing its various manifestations. The research interest in them is rooted in the fact that OCB and its associated phenomena affect role behavior and work performance, cost reduction [8] and employees’ adaptation to organizational changes [9], etc. Attention is also paid to the different antecedents of OCB, among which various identifications of workers, e.g., identification with an organization or identification a with small work group [10,11], play an important role.

Because the identifications and citizenship behavior of workers in an organization are multidimensional constructs, research on them should take into account: (a) the levels (focuses) of identification (e.g., organizational or group identification) and their components (i.e., cognitive and affective), and (b) the types (e.g., behavior oriented toward others) or specific dimensions of OCB (helping behavior, voice behavior, etc.). In addition, it is necessary to assess the OCB not only in general but also in a certain structural context: an informal subgroup, a small work group, a division or an organization as a whole. Only the consideration of all of these variables makes it possible to accurately assess and understand in detail the relationship “identification–OCB”.

### 1.1. Dimensions of Identification and Citizenship Behavior of Employees in an Organization

#### 1.1.1. Identification Levels and Components

In large and even medium-sized organizations, the following structural levels can easily be recognized: (1) the organization as a whole, which can be either an independent unit (for example, a higher education institution) or a subsidiary (a branch) of a large corporation; (2) the secondary-level structural unit or division (for example, a department within a university or a specific manufacturing facility within a company); (3) a small group, i.e., a basic-level structural unit, which is usually included in a secondary-level unit (for example, a laboratory within a university department or a self-regulating small work group in a manufacturing facility). These structural units serve as identification foci, and employees accordingly develop organizational identification (organizational ID/OID) [12,13], sub-organizational identification (sub-organizational ID/SoID) [14,15], or group identification (group ID/GID) [16,17].

In addition, relatively stable and situational informal subgroups spontaneously emerge within small work groups. In each stable subgroup, the members are integrated based on common psychological characteristics that are important to them and, in turn, distinguish them from other members of the group. Subsequently, there may be another structural level, i.e., an informal subgroup, which many workers can identify themselves with. This identification type can be denoted by the term “micro-group identification” (micro-group ID/MgID) [18]. In this study, we only examine workers’ identification with relatively stable subgroups within their work group, and not with the intergroup (cross-group) informal subgroups (i.e., those that include members from different work groups and departments). Together, these four identifications are encompassed by a common concept of “social identification”. This is consistent with the idea that people may have as many social identifications as there are groups to which they feel they belong [19]. Interestingly, micro-group identification, in particular, was found to be stronger for most group members than their group identification [18], whereas the latter is quite often stronger than organizational identification [20,21]. However, social identifications vary in subjective significance for a person, in terms of their stability and manifestations in different situational contexts.

Workers can also identify themselves with a leader/supervisor [22,23] or with peers, i.e., individual colleagues [18,24] within their work group. In such cases, “interpersonal identification” (interpersonal ID/IID) is indicated. In addition, all individuals have a personal identification (personal ID/PID) [25], of which the strength and degree of manifestation can vary depending on both individual and contextual characteristics.

Some researchers understand social identification as a multidimensional construct which includes several components, e.g., cognitive, affective, evaluative and behavioral components [26]. The literature has not yet fully sorted out the issue of identification components. The cognitive and affective components are most common, while other components are still debated. For example, the evaluative component can be understood as part of the affective component, because any emotional experience of feeling something has an evaluative component. Because identification is a certain relationship of an individual to some subject (person, collective), a particular associated behavior is more likely a consequence of that relationship, rather than its component. This argument is consistent with the idea that behavior is a likely result of identification, and not its component [27]. In our opinion, identification components, e.g., cognitive and affective components, are essential characteristics not only of any social identification (including the above-mentioned OID, SoID, GID, or MgID) but also of interpersonal and personal ones.

**Figure 1:**
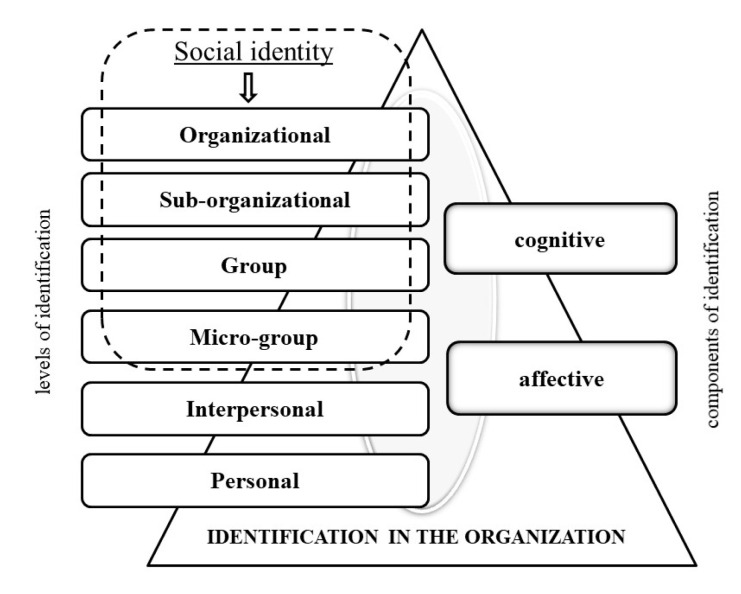
Multidimensional conceptual model of employees’ identifications in an organization.

Therefore, we propose a multidimensional conceptual model of employees’ identifications in an organization (Figure 1) which is based on two factors: (1) levels of identification (depending on the foci of the identification), i.e., organizational, sub-organizational, group, micro-group, interpersonal and personal; and (2) components of identification (depending on the content of the identification), i.e., cognitive and affective. Identification (encompassing all of the above-listed levels and components) is the process of an individual experiencing and feeling their engagement with a significant subject (individual or collective) and their connection with it, as well as the perception of oneself or another in accordance with his/her actual characteristics, as well as reliving events happening to him/her [28]. All identifications, including personal ones, are based on the connection of an individual with self or others. We do believe that by taking into account all of the dimensions (both levels and components), a more comprehensive picture of employees’ identifications in an organization can be construed, and their influence on OCB and associated behaviors can be more thoroughly examined.

#### 1.1.2. Dimensions and Types of OCB

The research literature most often presents OCB as a generalized (composite measure) construct. However, some studies have examined its specific dimensions, such as conscientiousness, sportsmanship, helping and civic virtue [29]. Several typologies of OCB have been proposed to systematically account for a whole variety of behaviors: (1) OCB-I, a behavior directed toward other individuals, and OCB-O, a behavior directed toward an organization [30]; (2) AOB, an affiliation-oriented behavior, and COB, a challenge-oriented behavior [5]; (3) a behavior oriented toward (a) self-performance (for example, personal industry, overtime work), (b) other individuals (providing help and support, cheerleading), (c) relationships (courtesy, peacekeeping), (d) work groups/units, divisions and/or entire organizations, the improvement and promotion of the corresponding structures (offering quality ideas and suggestions, civic virtue), and (e) maintaining (compliance with/adherence to) rules and regulations [31]. The third typology is more differentiated than the other two, and it allows for a clearer discrimination among the classes of OCB dimensions. The current study explores two specific behaviors: (1) offering quality ideas and suggestions and (2) providing help and support. The former refers to behavior oriented toward improving and promoting the small group, whereas the latter depicts behavior oriented toward other individuals.

### 1.2. Relationship between the Dimensions of Identification and OCB

#### 1.2.1. Gaps in the Study of ID–OCB Connections

In order to review and summarize what is known about ID–OCB relationships, we searched for relevant empirical studies published between 1995 and April 2019 in the following databases: Academic Search Complete (EBSCO), Business Source Complete (EBSCO), PsycINFO and PsycArticles (APA PsycNET), and in additional sources. These searches resulted in 81 publications containing 96 independent studies that featured 149 ID–OCB correlations. We only considered empirical studies, of which the samples included employees of government and commercial organizations, in which valid instruments (i.e., standardized or otherwise proven to reflect the constructs in question) were used. In summary of these studies’ findings, several important circumstances should be noted. First, organizational ID was the most frequent correlate of OCB (112 correlations), substantially surpassing in frequency all of the other correlates, namely: group ID (20), interpersonal ID (11), sub-organizational ID and micro-group ID (three correlations, each). Obviously, empirical studies disproportionally focus on organizational ID, whereas studies of other identifications are rather underrepresented. At the same time, no research reported the degree of connection between personal ID and OCB.

Second, only two publications differentially assessed the components of identification (e.g., cognitive and affective) and their contribution to OCB [26,28]. It turned out that the components of a given identification could contribute to certain dimensions of OCB, not necessarily in the same way.

Third, in 40% of the reviewed correlations, OCB was measured as a generalized construct or, in other words, as a composite measure [13,32]. In 19% of cases, the OCB types were the subjects of differentiated measurement, for example, OCB-I and OCB-O [10,33], or AOB and COB [14,34]. In other cases, specific OCB manifestations were assessed, e.g., courtesy and sportsmanship [26], helping behavior [16], civic virtue [35], voice behavior [36] and taking charge [22]. We believe that assessing generalized OCB and even some types of dichotomous typologies (e.g., OCB-I and OCB-O) could lead to over-generalization. Therefore, it may be advisable to assess more specific OCB dimensions, or to rely on a more differentiated OCB typology (for example, behavior oriented toward the performance of one’s own work, toward other people or relationships, etc.).

Fourth, only seven studies assessed OCB exclusively in the context of a small work group, in two studies at the level of a secondary organizational unit, whereas in all other cases the assessment was at the level of the organization as a whole. However, it must be taken into account that in large and even medium-size organizations, most frontline employees exhibit OCB (e.g., helping, voice) primarily within a small work in-group, rather than at the level of a division or the entire organization. This happens largely because of the specific characteristics of an employee’s activity in these organizational strata. That is, the professional activity of most employees takes place mainly in the group (department, unit, shift, etc.), they have higher interdependence with colleagues within the group than with members of out-groups in the organization, and their work responsibilities concentrate primarily within the group. Therefore, it would be more logical to measure such behaviors primarily in the context of a small group.

#### 1.2.2. Connection of Specific IDs with OCB

Our further reasoning is based on these facts, and thus largely focuses on OCB in the context of a small work group. In addition, we will take into account the degree of regularity (e.g., daily or less often) and intensity (the duration and consistency of engagement) of interactions among the employees, primarily within their respective in-groups. These characteristics of interactions depend on particular work tasks and conditions. Interdependent tasks determine a higher intensity of interaction (most of the work is performed mainly by the group members, such that the acts of interaction are an essential part of it and relatively long), whereas more autonomous tasks determine a weaker intensity of interactions. When employees work full time and are together in the same workspace, the interactions are regular. If working hours are irregular/flexible, the interactions among workers are often irregular as well. As such, inside the research lab, production team, law enforcement patrol squad, or control room operational shift, etc., the regularity of interactions is much stronger than among the faculty of a department of a higher education institution or among agents/guides in a small travel company (for more details on the working conditions of academic workers in Russia, please see the Sample subsection.)

It is logical to assume that group ID (as compared with organizational ID) is a stronger predictor of many dimensions of OCB within a group. This consideration is supported by the results of a meta-analysis that compared the associations of organizational and group attachment (identification and commitment) with OCB at the organizational and group levels [20]. It was found that group attachment, as opposed to organizational attachment, correlated with extra-role behavior to a larger degree at the group level than at the level of the entire organization. However, it is not clear which factor—identification or attachment—played a leading role in this association. There may be a significant positive association of organizational ID with OCB in a group, depending on a number of circumstances. For example, the smaller the organization, or the more open the group is to the organization, or the closer the collaboration between groups within the organization, the stronger this association would be. The more regular and intensive the interactions of the employees with each other, not only in the group but also beyond it, in the context of the unit and/or the entire organization, the more pronounced such a connection should be. However, even then, this connection will either be evident just for a single component of identification (not both), or will still be weaker than the relationship of the group ID or micro-group ID with the OCB in the context of the work group.

At first glance, there should also be a positive relationship between interpersonal ID (i.e., identification with colleagues) and micro-group ID, on one hand, and OCB in the work group, on the other, and some research confirms just that [24,37]. However, it is not a universal trend, as these types of interconnections may depend on various contextual characteristics, for example, on the high or low regularity and intensity of interactions among group members, which, in turn, are contingent on the specific nature of the work that the group performs. In work groups characterized by a low regularity of interactions, colleagues identify less with each other within the group. In such groups, the process of informal subgroups’ formation is not very salient. Therefore, micro-group identification there is typically weaker. As a result, groups with a low intensity of interactions are less likely to associate interpersonal ID and micro-group ID with OCB. On the contrary, such a connection may well be observed in groups with high levels of regularity and intensity of interactions.

In light of all of the above, the following research hypotheses were formulated to guide this study:

**Hypothesis** **1** **(H1).** *Under the conditions of regular and intense within-group interactions:*

**Hypothesis** **1a** **(H1a).** *Organizational ID is positively associated with both offering quality ideas and suggestions and providing help and support in small work groups.*

**Hypothesis** **1b** **(H1b).** *Group ID is positively associated with both offering quality ideas and suggestions and providing help and support in small work groups.*

**Hypothesis** **1c** **(H1c).** *Micro-group ID is positively associated with both of these OCB dimensions in small work groups.*

**Hypothesis** **1d** **(H1d).** *Interpersonal ID is positively associated with both OCB dimensions.*

**Hypothesis** **2** **(H2).** *Under the conditions of irregular and weak interactions, only group ID, in comparison with other identifications, has a significant positive relationship with both dimensions of OCB under consideration: offering quality ideas and suggestions, and providing help and support in small work groups.*

#### 1.2.3. Interaction Effects of Group ID and Other IDs, and Their Connections to OCB

Some identifications can create interactive effects on civic behavior. It has been found that both strong group ID and organizational ID are more closely associated with extra-role behavior than when only one of the two identifications is strong [38]. However, another study has shown that group ID and organizational ID individually are positively associated with OCB, but jointly create a negative effect on extra-role behavior [39]. We believe that the very idea that the ratio of identifications (group ID and organizational ID) may have effect on behavior should be extended to other levels (foci) of identification, such as micro-group ID, interpersonal ID and personal ID.

We assume that the role of the relationship of social identifications (organizational, sub-organizational, group and micro-group) in OCB depends on a number of conditions, for example, on the organizational structure (organization, department, group or subgroup) in which the OCB appears. When employees demonstrate OCB primarily within a group, group ID is more likely to take the lead. It is more relevant to the civic behavior of workers in the in-group. Therefore, group ID should be taken as a basic one, and its ratio with other identifications should be analyzed. Thus, the ratio of group ID and organizational ID may be a predictor of some dimensions of OCB in an in-group. Moreover, the stronger the group ID and the weaker the organizational ID, the stronger certain OCB dimensions will be in a group. Under the conditions of a high regularity and intensity of interactions, such a connection can extend to many types of OCB, including behaviors focused on improving group performance (e.g., offering quality ideas and suggestions), as well as behaviors focused on other people (e.g., providing help and support) in a group. However, in environments characterized by a low regularity and intensity of interactions, this kind of association would extend rather to performance-oriented behaviors (e.g., offering quality ideas and suggestions) than to any other OCB type.

The ratio of group ID and micro-group ID can also be associated with some behaviors. However, the type of connection will be different in groups with a high and low regularity and intensity of interaction. In groups with a high regularity of interaction, stable informal subgroups are more clearly formed, which play a significant role in the life of the group. A member included in a subgroup is more strongly identified with his subgroup than with the group as a whole. Even some members who are not included in subgroups can strongly identify with certain subgroups that are close (referent) for them. As a result, the behaviors (including the citizenship ones) of workers with a strong micro-group ID are often determined by the priorities of their informal referent subgroups, and not by their personal or group interests. Therefore, it can be assumed that the weaker the group identification and the stronger the micro-group ID, the more frequent and stronger the behavior focused on improving the performance of the group and the behavior focused on other people will be, and vice versa. In groups with a low regularity of interaction, the formation of informal subgroups is not so obvious, and therefore they do not play a significant role in the life of the group and its members. Consequently, in such groups there will be a different connection: the stronger the group ID and the weaker the micro-group ID, the stronger certain behaviors will be, and vice versa. This trend is more likely to affect behaviors oriented toward improving group performance and behaviors oriented toward other people, or interpersonal relationships in the group characterized by a high regularity and intensity of interactions. In turn, under the conditions of a low regularity and intensity of interactions, such a connection appears to be more typical for behaviors focused on improving group performance than for behaviors focused on other people.

The ratio of group ID and interpersonal ID will affect behavior oriented towards promoting groups, rather than behavior oriented towards other people in the group. This type of the connection will be characteristic for groups with a high degree of regularity and intensity of interactions. That is, the stronger the group ID and the weaker the interpersonal ID, the stronger the first type of behavior will be, in particular quality ideas and proposals.

It is also necessary to take into account the ratio of group ID and personal ID as a possible determinant of OCB. There are two points of view on the ratio of social (including group) and personal identifications. According to the first, they are mutually exclusive [40], and the second suggests their complementarity, i.e., their equality and complementarity [41], or their simultaneous manifestation [42]. Both points of view can take place depending on a number of circumstances, for example, depending on the characteristics of professional activity and institutional specifics. The more institutionalized the collective, the lower the freedom of expression of personal identity will be [43]. In this case, an increase in antagonism between personal and social (group and organizational) identifications is likely to be characteristic. In turn, creative professions (for example, academic works) presuppose the manifestation of individuality and, as a result, a stronger personal identification based on professional characteristics. In this case, the weakening of the incompatibility of the two identifications is possible. It will be more obvious the more democratic the environment in the organization. Therefore, we can assume that a relationship exists between the ratio of group ID and personal ID, on one hand, and the two OCB types under consideration, on the other. The stronger the group identification is and the weaker the personal identification is, at the same time, the more apparent this connection should be. This tendency would, more likely, manifest itself under the conditions of highly regular and intense interactions.

**Hypothesis** **3** **(H3).** *Under the conditions of a high degree of regularity and intensity of interactions:*

**Hypothesis** **3a** **(H3a).** *The ratio of group ID to organizational ID predicts both OCB dimensions under consideration: offering quality ideas and suggestions, and providing help and support (a positive association of the GID/OID ratio with both behaviors is expected).*

**Hypothesis** **3b** **(H3b).** *The ratio of group ID to micro-group ID predicts both OCB dimensions under consideration (the negative association of the GID/MgID ratio with the corresponding behaviors is expected).*

**Hypothesis** **3c** **(H3c).** *The ratio of group ID to interpersonal ID predicts the offering quality ideas and suggestions OCB (the positive association of the GID/IID ratio with this behavior is expected).*

**Hypothesis** **3d** **(H3d).** *The ratio of group ID and personal ID predicts both OCB dimensions under consideration (the positive association of the GID/PID ratio with either behavior is expected).*

**Hypothesis** **4** **(H4).** *Under the conditions of a low degree of regularity and intensity of interactions:*

**Hypothesis** **4a** **(H4a).** *The ratio of group ID to organizational ID predicts the offering of quality ideas and suggestions (the positive association of the GID/OID with this OCB dimension is expected).*

**Hypothesis** **4b** **(H4b).** *The ratio of group ID to micro-group ID predicts the offering of quality ideas and suggestions (the positive association of the GID/MgID ratio with this behavior is expected).*

**Hypothesis** **4c** **(H4c).** *The ratio of group ID to personal ID predicts both dimensions of OCB under consideration (the positive association of the GID/PID ratio with the corresponding behaviors is expected).*

### 1.3. Organizational Communicativeness Moderates the Connection between Identifications and Citizenship Behaviors

For a productive discussion of emerging problems and their possible solutions, and of the ways of performing tasks efficiently and making decisions in the work group, some characteristics of communications among employees may play particularly significant roles. We would name, as one of the most important features of business/instrumental interaction among employees, their organizational communicativeness. Concepts such as an organizational communication network [44] and internal (intra-organizational) communication [45] are quite well known in the research literature. The former is characterized by the following distinctive features: reliance on pre-existing roles and adherence to an efficient, heavily coordinated structure. The latter is defined as formal or informal messages sent and received through the organization. However, the construct of “organizational communicativeness” (OC) has a different meaning and reflects certain attitudes and behavioral skills. Namely, OC is the employees’ willingness and ability to exchange information and interact with each other in the process of performing tasks and professional roles. OC encompasses: (a) the transfer of business information, i.e., employee’s readiness and ability to share the information necessary for work with colleagues; (b) understanding of opinions, i.e., the readiness and ability to find mutual understanding with other members of the work team on the business issues being discussed, as well as their willingness to admit mistakes/shortcomings and the correctness of the alternative opinions of colleagues; and (c) the coordination of actions, i.e., the readiness and ability to act in concert with others in order to coordinate and combine efforts to solve common problems. The high organizational communicativeness of an individual employee can strengthen her/his cooperative behavior and contribute to group activities, etc. Combining both strong group ID and high organizational communicativeness can, with high probability, enhance behaviors oriented toward group promotion. Thus, the instrumental communication skills of an employee can create a direct positive effect on the specified type of OCB, particularly offering quality ideas and suggestions.

**Hypothesis** **5** **(H5).** *Organizational communicativeness moderates the association between Group ID and offering quality ideas and suggestions, independently from the degree of intensity and the regularity of interactions within work groups.*

To summarize, the main objective of the current project is to study the association between identifications (personal, interpersonal, micro-group, group and organizational) according to both their components (cognitive and affective), on one hand, and to two dimensions of OCB, i.e., offering quality ideas and suggestions and providing help and support in small work groups, on the other.

## 2. Materials and Methods

### 2.1. Sample

This research was conducted on two samples in Russia during the year 2019 until the beginning of 2020. The first sample (Study 1), characterized by a relatively high regularity and intensity of interactions among employees, was composed of workers from eleven large and medium-sized commercial enterprises, representing various industries: mechanical engineering, dairy product manufacturing, oil refining, car retail, real estate, construction, and information technology, as well as state-owed regional institutions providing social services to the population (*N* = 183). The sample were 35.0% men and 65.0% women, aged 24 to 61 (*M* = 37.23; *SD* = 8.28), whose average tenure in their respective organizations/structural divisions was 5.72/4.79 years, respectively. These organizations’ structure include small groups composed of members with predetermined roles and responsibilities according to the staffing registry. These employees are constantly present at their workplaces throughout the workday/shift, and therefore they regularly interact among themselves within the group with a relatively high intensity.

The second sample (Study 2), characterized by a relatively low regularity and intensity of interactions among the employees, was formed of the academic staff of seven public higher education institutions (*N* = 157). The participants in this sample (34.4% men and 65.6% women) were aged from 23 to 75 (M = 44.19; SD = 11.94), and their average work experience at the university/department was 13.82/11.01 years, respectively. All of the participants were full-time (tenured) employees of the corresponding departments (22 departments in total), i.e., they were members of the respective small work groups. As in the vast majority of higher education institutions in Russia, the academic workers in this sample are not required to be at the workplace during the entire workday, but only to conduct lectures, seminars and consultations with students according to the departmental schedule, as well as to participate in department meetings and some other, rather infrequent/irregular events. The rest of the time, academic staff typically work at home and other locations, for example, conducting research and preparing teaching materials. Therefore, most employees do not interact regularly and intensively.

The two samples did not differ in gender composition, with a nearly identical proportion of women and men in both. However, there were some differences in the average age and work experience (length of stay in the organization), namely that both indices were higher for the academic workers than for the employees of commercial enterprises. These observed patterns are typical for the corresponding samples in the Russian context. Academic workers there undergo a competitive selection process and then sign fixed-term employment contracts with the head of the respective higher educational institution, usually for a period of five years. After the completion of that contract, the chances for success in the subsequent tender for a new contract are quite high. The vast majority of workers in academia (not just professors and lecturers) prefer to keep their jobs, as finding academic work at another institution in the same city (where they live) is very difficult, whereas the economic mobility for most of the population is still very low. As a result, the rejuvenation of academic staff is slow. Therefore, this category of employees typically has longer work experience and a higher average age than the employees of commercial companies, which are characterized by much higher labor mobility.

### 2.2. Measures

Four instruments were used for the assessment of the identifications: (1) an Organizational Identification Questionnaire (OIQ); (2) a Group and Micro-group Identification Questionnaire (GMGIQ); (3) an Interpersonal Identification Questionnaire (IIQ); and (4) a Personal Identification Questionnaire (PIQ) [28]. Each questionnaire consisted of six items: three per subscale of the cognitive and affective components of the respective identification. Also, the GMGIQ instrument contained two sections entitled “Work group/unit” or “Department” (for the measurement of group identification) and “Community of colleagues with whom I maintain friendly relations” (for the assessment of micro-group identification). The items in both sections were identical, but applied to group ID and micro-group ID. All of the questionnaires utilized a six-point response scale (from “1”—“strongly disagree” to “6”—“strongly agree”). The following values of Cronbach’s α characterize the scaled across the set of the instruments used. For the scale of “cognitive identification”, they were: 0.75 (OIQ); 0.75 and 0.74 (GMGIQ, for its two sections, respectively); 0.78 (IIQ); and 0.83 (PIQ). For the scale of “affective identity”, these values were: 0.78 (OIQ); 0.81 and 0.79 (GMGIQ); 0.79 (IIQ); and 0.71 (PIQ). Here are some examples of items which composed the scale of cognitive identification: “I sense I am a part of the whole organization” (OIQ), “I perceive common successes or failures as my own” (GMGIQ), “Often I think the same way as some other colleagues” (IIQ), “I feel that I perceive a lot of things differently from how most of my colleagues perceive them” (PIQ). For the scale of affective identification, the examples are: “As a rule, I am pleased to realize that I work in this collective” (OIQ), “As a rule, I am pleased to be a part of my particular” (GMGIQ), “I feel frustrated, when I cannot reach mutual understanding with those who interest me” (IIQ), “Often I am more concerned about my own successes than about successes of my colleagues or the of team” (PIQ).

Furthermore, the study employed a two-factor questionnaire of organizational citizenship behavior in a work group, which featured the following subscales (each composed of three items): (1) offering quality ideas and suggestions (for example, “Inclined to give a comprehensive and thoughtful assessment problems, situations or state of affairs”), and (2) providing help and support (for example, “Help colleagues when they ask for it”) [46]. These subscales measure behaviors that fall under different OCB categories. The first subscale measures behavior that is categorized as “orientation towards improvement and group advancement”, whereas the second measures behavior that falls under the category of OCB “orientation toward other people”. This is the peer-review type of a questionnaire, where the leaders of work groups (supervisors/managers) serve as the reviewing experts. They rated the two corresponding dimensions of within-group OCB of their employees on a 6-point scale: from “1” (“strongly disagree”) to “6” (“strongly agree”). The Cronbach’s α values were 0.80 and 0.79 for the first and second subscale, respectively.

The organizational communicativeness questionnaire reflects such aspects of professional interaction as the transfer of business information, understanding the opinions of colleagues and coordinating actions with colleagues within a work group [47]. It consisted of 6 items (for example, “Ready to share information necessary for job performance with colleagues” or “Can act in concert with colleagues when performing tasks”). The Cronbach’s α value was 0.90. Using this questionnaire, the managers rated their employees on the same 6-point scale as in the previous questionnaire. 

### 2.3. Procedure 

The selection of organizations in the socio-economic sphere was carried out in two stages. First, we contacted the executives of a large number of companies with the brief outline of the intended study. Several enterprises and one institution providing social services to the population, agreed to conduct the survey among their employees. Subsequently, we were given access to the appointed work groups of which the members were asked to participate in the study. In the higher education institutions, we discussed the research plan with the heads of departments. They informed the academic staff of their respective departments about the research and invited them to take part in it. Before becoming study participants, all of the respondents were informed about its objectives and consented to participate. With the approval from the administration of the corresponding companies/institutions, the study was carried out at the workplaces. The participants completed the above-described questionnaires on the paper forms distributed and then collected by the researcher.

### 2.4. Data Analysis

The data analyses included the calculation of the descriptive statistics and Spearman correlation coefficients, as well as a bias-corrected bootstrapping technique. The analytical procedures were carried out using and statistical software package *SPSS 23,* including *PROCESS-macro* (Model 1) within it.

## 3. Results

### 3.1. Connection between Identifications and OCBs

In order to test the first two hypotheses (H1 and H2), Spearmen correlation coefficients between the identifications (for cognitive and affective components separately) and the two measurements of OCB were calculated (Table 1).

In Study 1 (the sample characterized by a relatively high regularity and intensity of interactions), organizational ID (by its affective component), group ID, micro-group ID and interpersonal ID (by both components) were significantly positively associated with the two investigated dimensions of OCB. Therefore, hypotheses H1a, H1b, H1c and H1d were confirmed. However, there were some exceptions: interpersonal ID was significantly correlated with offering quality ideas and suggestions only by the cognitive component, and organizational ID was significantly correlated with the same OCB only by the affective component. In Study 2 (the sample characterized a relatively low regularity and intensity of interactions), the cognitive component of group ID was significantly positively associated with just one of the studied OCB dimensions, i.e., offering quality ideas and suggestions, whereas its affective component was significantly positively associated with both dimensions of OCB. The remaining identifications showed no significant correlations with either of the OCB dimensions. Therefore, H2 was confirmed.

### 3.2. Ratio of Identifications and OCB

In order to test Hypotheses 3 and 4, the indices of the personal, interpersonal and organizational identifications were initially standardized and converted into points on an interval scale (sten scores). The micro-group ID and group ID data were not standardized, as they were measured on exactly the same list of items. Furthermore, the indicators of the ratios of group ID with all of the other identifications were calculated, i.e., with the numerical value of group ID in the numerator, and the value of each other identification in the denominator, sequentially. It was assumed that the stronger the group identification and the weaker the other identification (e.g., organizational), the more likely a higher positive relationship between the ratio of the two identifications and the measure of the OCB dimension in the context of a work group will be. The most indicative, in a mathematical sense, is a ratio between group and any other identification (across individual participants) greater than ‘one’. The only exception appears to be the ratio of group ID to micro-group ID in Study 1, in which the negative connection is presumed. Conversely, the ratio of a weaker group ID and a stronger other identification (less than ‘one’ in value) is highly likely to be negatively associated with the particular manifestation of OCB. After these data adjustments, Spearman correlation coefficients were calculated between the produced ratios and both OCB dimensions (Table 2). As a tool from the arsenal of nonparametric statistics, this type of correlation does not require the meeting of the assumption of normality for the distribution of the correlated variables. In addition, this analysis assumes that if you swap the values in the numerator and denominator (e.g., move around group ID and organizational ID), then only the sign of the corresponding coefficient of correlation will change (from positive to negative, or vice versa). This method of analysis seems to be suitable for testing Hypotheses 3 and 4.

The most convincing confirmation was observed for the H3d (Study 1), as significant positive associations of the group ID/personal ID ratio (for both cognitive and affective components) with either of the OCB dimensions. The same group ID/personal ID ratio, but only for the affective component, was significantly positively associated with both dimensions of OCB in Study 2. It also confirmed H4c for one identification component. H3a and H4a were confirmed for only one component of the identification. Specifically, the group ID/organization ID ratio (for the cognitive component) was significantly positively correlated with the OCB of offering quality ideas and suggestions (Study 1 and 2) and with the OCB of providing help and support (Study 1).

The group ID/micro-group ID ratio for the cognitive component of these identifications was significantly negatively associated with both dimensions of OCB in Study 1, and for their affective component, it was significantly positively associated with offering quality ideas and suggestions in Study 2. Therefore, H3b and H4b were confirmed, but only for one of the two components of the corresponding identifications. H3c was confirmed for the affective component: the Group ID/Interpersonal ID ratio was positively associated with offering quality ideas and suggestions in Study 1.

### 3.3. Moderating Effect of Organizational Communicativeness

We tested the role of organizational communicativeness in the relationship between group ID (by both cognitive and affective components) as independent variables, and the two dimensions of OCB as dependent variables. In order to analyze the effects of moderation, we used a bias-corrected bootstrapping approach. This analysis was performed with the PROCESS macro (Model 1) using the *SPSS-23* software package.

The results of the regression analyses are presented in Table 3. None of the models in either Study 1 or Study 2 revealed any significant moderating effect of organizational communicativeness. As such, H5 was not confirmed. However, as reflected in Table 1 above, organizational communicativeness is significantly positively associated with the two OCB dimensions in both samples (Study 1 and Study 2), which could indicate that organizational communicativeness might predict these behaviors, regardless of the intensity and frequency of the interactions.

## 4. Discussion

In our study, most of the hypotheses were confirmed. Moreover, some of these results, when compared with the findings of other research, reveal rather interesting patterns. For example, we found significant positive correlations between group ID and the two measures of OCB within the groups in Study 1 and 2. These findings: (a) agree with the research that showed group ID has a significant association with the providing help and support to the colleagues OCB type [26] and with OCB-I [17] within a group; (b) contradict some other studies in which this identification did not significantly correlate with either helping behavior [16] or COB [34]. The positive significant association between identification with coworkers (interpersonal ID) and the two dimensions of citizenship behavior in Study 1 is consistent with the data from some other studies, where this identification was positively correlated with helping behavior [24] and contributions to collaborative group activities [37]. Similarly, the relationships between micro-group ID and OCB in Study 1 confirm the result observed in a different sample. Significant associations, shown in both Study 1 and Study 2 between the ratio of group ID and organizational ID, on one hand, and offering quality ideas and suggestions, on the other, are consistent with the data from A.A. Klimov [39], but are not in line with the results of R. van Dick et al. [38]. These and similar comparisons are relative, as the related studies varied in terms of the participating samples; in particular, they did not separate their respective samples by the degree of intensity and the regularity of interactions. Furthermore, many did not take into account the cognitive and affective components of the identifications being studied, and only used participants’ self-reports as assessment tools. The rest of our results, especially with regard to the confirmed hypotheses, are rather difficult to compare with the results of other studies, simply because we were not able to locate research that examined these types of questions. When stating the hypotheses for the study, we shared the rationale behind them based on our review of previously conducted research in the field, such that the observed results, by and large, are explained and supported by that reasoning. In order not to repeat ourselves, we will briefly discuss only the main (most consequential) results. First, the degree of intensity and regularity of interactions affects the relationship of identifications (except for the personal and group ones) with both dimensions of OCB at the level of a small work group. Specifically, in groups with a relatively low regularity and intensity of interactions (Study 2), in contrast to groups with a high regularity and intensity of interaction (Study 1), interpersonal ID and micro-group ID are notably weaker. As a result, in such groups, there was virtually no connection between interpersonal ID and micro-group ID, on the one hand, and the two OCB measures, on the other. Group ID significantly predicts OCB regardless of the interaction characteristics. We attribute this to the assumption that the real group membership is one of the important conditions for forming a group ID and manifesting OCB within a group context. Therefore, even in the case of irregular interactions in groups, the group ID would be strongly reflected in OCB. Second, the study results confirmed the assumption that there are interaction effects for some IDs with respect to OCB. Most of all, this concerns the relationships of group IDs with other identifications. The interaction effects can depend both on the components (cognitive and affective) of identification, and on the characteristics of the interactions (regularity and intensity) in groups. Getting away from the minor details, we found that the stronger the group ID is and the weaker the other identifications (personal, interpersonal and organizational) are, the stronger certain OCB dimensions could be. This tendency depends on the degree of regularity and intensity of the within-group interactions rather feebly. However, the relationship between the ratios of group ID to micro-group ID and OCB does depend on the specified interaction characteristics. In other words, in groups with a low regularity and intensity of interaction, the stronger the group ID and the weaker micro-group ID, the stronger the OCB is manifested in the work group. In turn, in groups with a relatively high regularity and intensity of interactions, the combination of weaker group ID and stronger micro-group ID is significantly correlated with both measures of OCB. We would argue that this pattern of results is due to the fact that, in such groups, stable informal subgroups are clearly formed and include a significant number of group members. Those included in informal subgroups tend to more strongly identify themselves with their respective subgroups than with the group as a whole. Even members not directly involved in subgroups can still strongly identify with certain subgroups that appear close to them (reference groups). Therefore, the OCB of employees with strong micro-group ID in certain situations is determined to a larger extent by the goals and interests of the reference subgroup, and not by personal or group goals and interests.

### 4.1. Theoretical Implications

The results of the study broaden our understanding of the relationships between different employees’ identifications within an organization and the OCB dimensions. They also shed light on the relationships between the ratios of group identification to other identifications and OCB in small groups, taking into account in particular: (a) identification components, and (b) the degree of the intensity and regularity of interaction in the respective groups. Previous research has generally focused on the links of just one type of identification (usually organizational) or, at best, two identifications (e.g., organizational and group) with OCB. In this study, we tried to address a wide range of identifications with individual and collective subjects in order to uncover the specific connections they have with the two selected dimensions of OCB. There have been only sporadic studies that have examined the effects of the ratios of different identifications on their associations with OCB. Moreover, their results were rather inconsistent and contradictory. The significant connections between the ratios of group ID to other identifications and the OCB dimensions under consideration and found in our study are indicative of the following: in a sense, antagonistic relationships exist between group ID and other identifications in terms of their relationships with particular OCB dimensions. This could be clearly observed, for example, in relation to the group ID/personal ID ratio (Study 1). Incidentally, the relationships of personal ID and micro-group ID with OCB have not actually been studied before. Our research demonstrated that the second identification was significantly correlated with the two selected OCB dimensions (Study 1), and that both identifications in their ratios with the group ID appear to be predictors of citizenship behavior.

In previous studies, with a few rare exceptions, the identification components (e.g., cognitive and affective) were not identified, separately measured or analyzed. However, as shown in our study, the roles they play in the connections between individual identifications, as well as the ratios of group ID to other identifications, on the one hand, and OCB dimensions, on the other, may be either quite similar or vary substantially. Furthermore, the vast majority of previous studies focused predominantly on the generalized concept of OCB or its dichotomous typology (i.e., OCB-I and OCB-O), at the expense of more differentiated OCB manifestations. In addition, OCB was rarely measured within the work group, but was predominantly assessed in the context of the entire organization. On the contrary, our study assessed specific behaviors related to different OCB types in the context of a small work group.

Additionally, we want to draw readers’ attention to the organizational communicativeness of workers, i.e., the concept that reflects their specific communication skills, which, as our study demonstrated, could be a strong predictor of the two OCB dimensions under consideration. The willingness and ability of an employee to share critical work-related information with colleagues, to find mutual understanding with them on important issues, and to join efforts for successful task completion create the essential foundation for: (a) cooperative behavior, (b) a significant contribution to group activities, (c) and helping and assisting other group members, etc. However, the effect of organizational communicativeness with respect to OCB can be moderated substantially by some personality traits (e.g., conscientiousness, agreeableness, responsibility) and by contextual variables (e.g., norms of cooperation and psychological climate in the group). Addressing these issues would be an interesting perspective for future research.

### 4.2. Practical Implications

Managers in an organization should be able to more confidently prognosticate the different nuances of employees’ behaviors (specifically, those addressed in our study: offering quality ideas and suggestions, and providing help and support within a small work group) if they are armed with the knowledge of: (a) the strength of employees’ particular identifications and their components, and (b) the ratios of group identification to personal, micro-group and organizational identifications. The other factor that quite clearly predicts these behaviors is organizational communicativeness. Based on all of this information, Human Resource Management could suggest reasonable measures aimed to strengthen certain identifications in order to increase the corresponding OCB dimensions. For example, positive differences between the in-group and outgroups could be identified and reinforced. Managers could give the group interdependent tasks, promote the goal of group success in task performance, and initiate competition between the group and other groups, etc. HR could also engage employees in various trainings designed to increase organizational communicativeness and, through this, contribute to strengthening particular citizenship behaviors.

### 4.3. Limitations and Future Research

In this study, we limited ourselves to two samples, from the socioeconomic sphere and higher educational institutions, respectively, and did not cover other professional areas with the corresponding strong or weak regularity and intensity of interactions. Furthermore, the current study examined the associations of different levels of employees’ identifications with only two OCB dimensions (offering quality ideas and suggestions and providing help and support) as typical representatives of the two major types of OCB: behaviour oriented towards the improvement and promotion of the group, and behaviour oriented towards other people. Therefore, in the future, it is necessary to test similar hypotheses, using more diverse samples that represent industries/organizations with both high and low regularity and intensity of interactions, as well as to study the connections of employees’ identifications with other dimensions of citizenship behavior, e.g., those that focus on personal work, relationships among people, and compliance with organizational rules and requirements.

The data collection for this research took place before the COVID-19 pandemic, so the sample in Study 1 (commercial enterprises and social services institutions) was truly characterized by relatively high degrees of frequency and intensity of interactions among employees. However, during the pandemic, some workers in a number of organizations may have been transferred to telecommuting and working at distance. As a result, in many small work groups, the intensity and frequency of interactions may have decreased. The same is true for a multitude of organizations outside of the scope of our research. These business arrangements, if proven reasonably efficient, may persist in the future. Therefore, further research in the area of relationships between identities and OCB should necessarily take into account these emerging work characteristics (specifically, a combination of attending a workplace in-person and remotely).

## 5. Conclusions

There are three major components to the uniqueness and actuality of this research: (1) It proposed a multidimensional (multilevel) model of employees’ identification in an organization. (2) Our research emphasized the need for a more differentiated approach to understanding organizational citizenship behavior, and also as a more complex phenomenon than many of the existing typologies consider. (3) Finally, in this paper we introduced and tested the idea that the ratios of various identifications could differentially affect their connections with OCB. These three components together enabled the more nuanced examinations of the relationships among different identifications (by two components: cognitive and affective), as well as the relationships of various identifications with the two selected dimensions of OCB in the context of small work groups.

## Figures and Tables

**Table 1:** Descriptive statistics of the major study variables and ID–OCB correlation coefficients.

Variable	Sample/Study	*M*	*SD*	1	2	3	4	5	6	7
1. PID	1	9.16/9.45	3.44/3.14							
	2	9.49/10.35	3.74/3.42							
2. IID	1	11.48/10.25	3.27/3.51	0.11/−0.17 *						
	2	12.54/11.03	2.58/2.94	−0.19 */−0.08						
3. MgID	1	12.88/13.37	3.64/3.83	0.07/0.04	0.41 ***/0.29 ***					
	2	13.23/13.95	2.64/2.86	−0.16 */−0.20 *	0.08/0.16 *					
4. GID	1	12.44/13.54	3.50/3.67	0.08/−0.04	0.43 ***/0.30 ***	0.78 ***/0.83 ***				
	2	12.82/14.14	3.22/2.92	0.01/−0.28 ***	0.18 */0.16 *	0.48 ***/0.67 ***				
5. OID	1	12.37/13.67	3.36/3.32	0.07/−0.01	0.34 ***/0.20 **	0.55 ***/0.66 ***	0.54 ***/0.72 ***			
	2	11.19/13.03	3.26/3.41	−0.10/−0.27 ***	0.13/0.17 *	0.28 ***/0.44 ***	0.26 **/0.51 ***			
6. QIS	1	12.24	3.50	−0.11/0.00	0.19 **/0.12	0.27 ***/0.35 ***	0.19 */0.32 ***	0.06/0.27 ***		
	2	13.11	3.15	0.01/−0.09	0.08/0.04	0.00/0.01	0.18*/0.17*	−0.09/0.07		
7. HP	1	13.00	3.86	−0.05/−0.04	0.28 ***/0.23 **	0.40 ***/0.51 ***	0.31 ***/0.43 ***	0.18*/0.40 ***	0.50 ***	
	2	13.46	3.39	−0.09/−0.09	0.11/0.05	0.03/0.13	0.05/0.21**	−0.07/0.10	0.59 ***	
8. OC	1	25.57	6.67	−0.04/−0.01	0.23 **/0.24 **	0.35 ***/0.45 ***	0.26 ***/0.41 ***	0.17 */0.37 ***	0.55 ***	0.69 ***
	2	27.18	6.06	−0.02/−0.10	0.13/0.07	0.10/0.21 **	0.11/0.30 ***	−0.14/0.11	0.66 ***	0.70 ***

Note: ID Levels: PID—personal identification, IID—interpersonal identification, MgID—micro-group identification, GID—group identification, and OID—organizational identification. Correlations by the cognitive component are reported before the slash, and those after it are by the affective component of the corresponding ID level. OCB dimensions: QIS—offering quality ideas and suggestions and HP—providing help and support. OC—organizational communicativeness. Sample: 1—Study 1 (Industry and Services, a relatively high regularity and intensity of interactions); 2—Study 2 (Higher Education, a relatively low regularity and intensity of interactions). *—p < 0.05; **—p < 0.01; ***—p < 0.001.

**Table 2:** Correlations between the ID ratios and OCB dimensions.

ID Ratio	OCB Dimensions			
	Offering Quality Ideas and Suggestions		Providing Help and Support	
	Study 1	Study 2	Study 1	Study 2
Group ID/Personal ID	0.25 **/0.28 ***	0.14/0.16 *	0.31 ***/0.35 ***	0.13/0.18 *
Group ID/Interpersonal ID	0.04/0.18 *	0.07/0.11	0.05/0.13	−0.03/0.14
Group ID/Micro-group ID	−0.21 **/−0.04	0.08/0.16 *	−0.25 **/−0.12	−0.03/0.10
Group ID/Organizational ID	0.17 */0.06	0.22 **/0.05	0.19 */0.02	0.12/0.06

Note: Values reported before the slash are correlations by the cognitive component, and those after it are correlations by the affective component of the corresponding ID level. *—p < 0.05; **—p < 0.01; ***—p < 0.001.

**Table 3:** Results of the regression analysis.

Variables	OCB Dimensions							
	Offering Quality Ideas and Suggestions				Providing Help and Support			
	Study 1		Study 2		Study 1		Study 2	
	M1	M2	M3	M4	M5	M6	M7	M8
Intercept	4.291	0.150	11.174	6.343	8.613	7.720	10.658	4.717
*Independent variables*:								
Group ID cognitive	−0.008		−0.549 *		−0.456 *		−0.592 *	
Group ID affective		0.368		−0.137		−0.348		−0.089
*Moderator variable*:								
Organizational communicativeness	0.303	0.424	0.022	.231	0.116	0.152	0.110	0.313
*R* ^2^	0.362	0.376	0.425	0.392	0.468	0.462	0.455	0.438
Δ*R*^2^	0.000	0.005	0.026	0.001	0.017	0.011	0.017	0.000
*F*	0.010	1.503	6.871 **	0.274	5.769 *	3.778	4.884 *	0.096

Note: *—p < 0.05; **—p < 0.01.

## Data Availability

The datasets generated during and/or analyzed during the current study are available in the Figshare repository (doi:10.6084/m9.figshare.14465742).
